# Apparent Trifles

**Published:** 1904-11

**Authors:** 


### APPARENT TRIFLES.

As to the apparent trifles which affect the comfort of the patient, the careful man sees to it that the chair is adjusted to the comfort of his patient as well as to his own convenience. A few minutes carefully spent in this seemingly unimportant detail will pay large interest on the investment. The patient will almost invariably thank you to make an application of some cooling emollent to the lips if they are at all dry or have a tendency to crack.

The rubber dam, if applied before excavating, will save much valuable time, though there may be room for question as to whether the average patient would not prefer to have its application delayed until the last moment. When ready to apply it there is room to demonstrate your ability to do things carefully. It will pay almost always to make six holes through the rubber and surround that number of teeth. I think the average dentist should have the dam applied to his own teeth by some careless man at least once a week. He would then use care in applying it for others. Ordinarily there should be no pain and very little discomfort through its application. In the cold weather, if you will place the clamp in the forceps, and then hold the clamp over your Bunsen just long enough to warm it, your patient will bless you. If in carrying your ligature between the teeth you are careful not to let it strike the gum suddenly, your patient will appreciate it. If you are about to fill an ordinary cavity it is absolutely cruel to ligate several teeth, carrying the ligature up above the natural gum line.

When preparing a cavity it is not necessary to have it so dry that the tears will come down the patient's cheeks because of the pain caused by the dryness and low temperature of the room as compared with the body. A pledget of cotton dipped in carbolic acid and passed through the lamp flame, then to the cavity, will change the expression on the face as if by magic. If applied cold the patient will dread it as much as the pain you seek to control. If when applying the dam clamp you fold two little pieces of muslin and lay them between the clamp and cheek, your patient will appreciate it.

If you are filling a large sensitive cavity with amalgam it will lessen the discomfort greatly if you will warm your plugger or burnisher each time you use either of them. If it is a gold filling you have made you will find much less complaint from your patient during the finishing if your bur is run into a bit of beeswax and your disk against a piece of soap or coated with vaseline before use. A corundum point is much less annoying if coated with vaseline instead of water, and answers as well when used on metal.—*International.*

